# Identification and Functional Analysis of Endophytic Bacteria *Bacillus cereus* in *Sphagnum palustre*

**DOI:** 10.3390/plants14101476

**Published:** 2025-05-14

**Authors:** Hongying Wang, Jiankang Xin, Xiaona Zhang, Shan Jiang

**Affiliations:** 1 College of Life Science, Guizhou Normal University, Guiyang 550025, China; 18286207392@163.com (H.W.); xinjiankang66@163.com (J.X.); 2 Research Center of Buckwheat Industry Technology, College of Life Science, Guizhou Normal University, Guiyang 550025, China; 3 School of International Education, Guizhou Normal University, Guiyang 550025, China

**Keywords:** isolation, functional properties, growth-promoting effect

## Abstract

Endophytic bacteria in *Sphagnum palustre* have a growth-promoting effect on plants. In this study, the endophytic bacterium strain J11 in *S. palustre* was isolated and identified as *Bacillus cereus*, and its growth cycle, functional characteristics, and effects on maize growth were analyzed. The results indicate that as *B. cereus,* the growth cycle of J11 consists of four phases, and the logarithmic phase lasts 2~24 h, with the abilities of phosphorus solubilization, protease, IAA, siderophore, and NH_3_ production. The phosphorus solubilization ability of J11 ranges from 1.66 ± 0.07 to 1.98 ± 0.07 mg/L, and the IAA production varies from 1.51 ± 0.07 to 8.67 ± 0.16 mg/L. It has a growth-promoting effect on maize by increasing the seed germination rate by 29.27%, plant height by 4.21%, leaf length by 17.12%, leaf width by 29.51%, above-ground fresh weight by 50.79%, below-ground fresh weight by 46.30%, and chlorophyll content by 56.81%. This study represents the first report on the isolation and identification of *B. cereus* from *S. palustre*. Furthermore, this study systematically investigated its multiple plant growth-promoting traits and functional characteristics. These findings provide valuable resources and a theoretical foundation for the development and functional exploration of microbial resources in agricultural applications.

## 1. Introduction

*Sphagnum,* a bryophyte belonging to the Sphagnaceae family [1], is a type of peat moss with significant economic value among moss species [2]. It is widely distributed, with over 300 species globally, including 47 species in China [3]. As a primary carbon-fixing plant in peatlands, *Sphagnum* has an annual carbon-fixation ability that surpasses any other genus of plants [4,5]. Covering only 3% of the Earth’s surface, it stores one-third of the global carbon source, making it an effective carbon sink [6,7]. Additionally, it plays a role in water conservation, hydrological regulation, and water quality purification, earning it the title of an “ecosystem engineer” [8,9,10]. In recent years, the multifunctional characteristics of *Sphagnum* have garnered increased attention with both ecological and agricultural sciences. For instance, Kostka et al.’s [11,12] study indicates that *Sphagnum* can facilitate carbon cycling. Hu et al.’s research highlights its ability to adsorb heavy metals such as chromium, zinc, and lead, thus aiding in soil remediation and environmental purification [13,14,15,16]. Previous studies by Gao et al. [17,18] demonstrated that *Sphagnum* plays a crucial role in regulating soil microbial communities, enzyme activities, and organic carbon content, thereby influencing soil fertility. Zhu’s [2] study further elucidates how *Sphagnum* improves seedling quality and vitality. *Sphagnum,* as an early terrestrial plant that needs to adapt to life on land, requires resilience against various biotic and abiotic stresses. Endophytes play a crucial role in this adaption by synthesizing bioactive compounds that help bryophytes withstand these stresses [19]. The mutual dependence between *Sphagnum* and its endophytes underscores their potential applications in agriculture and environmental protection.

Endophytes in *Sphagnum* refer to a group of microorganisms that survive partially or entirely within the moss without causing obvious infection symptoms in the host plant, including bacteria, fungi, and actinomycetes [19]. These internal bacteria in *Sphagnum* are of particular interest due to their functional diversity [20], adaptability, phosphorus solubilization [20], IAA production [20], nitrogen fixation [21,22], ability to modulate microbial communities via signaling molecules [23], and their antimicrobial properties [20,24]. Shcherbakov et al.’s study [24] indicates that strains of *Pseudomonas* and *Serratia* genera isolated from *Sphagnum* possess IAA-producing, hydrolase, and antibacterial capabilities. These bacteria colonize the rhizosphere of wheat and tomato plants, promoting their growth. Wang et al.’s research [21,25] further demonstrates that strains from the *Methyloferula* genus and nitrogen-fixing endobacteria from the *Burkholderia* genus can enhance *Sphagnum* growth by providing carbon and nitrogen. Currently, endobacteria identified in *Sphagnum* include strains from *Pseudomonas*, *Serratia*, *Methyloferula*, *Burkholderia*, *Flavobacterium*, *Collimona* [24], and *Methylocella* [26,27,28]. These species have shown growth-promoting effects on various plants. However, it remains to be further explored whether *Sphagnum* contains other growth-promoting bacteria.

*Bacillus cereus* Frankland, a Gram-positive, facultatively anaerobic bacterium belonging to the genus *Bacillus* (Bacillaceae) [29], exhibits a ubiquitous distribution across diverse environments including soil, water, dust [30], and various animal [31,32,33] and plant hosts [34]. This species demonstrates significant agricultural value, displaying both notable antagonistic activity against plant pathogens [35,36] and plant growth-promoting properties that enhance crop yields [36,37]. However, to date, no studies have reported the presence of endophytic *B. cereus* in *Sphagnum*.

In this context, the present study aims to isolate and identify endophytic bacteria from *Sphagnum palustre* L., investigate their growth curves, and assess their abilities in phosphorus solubilization, nitrogen-free environment growth potential, protease, IAA, siderophore, and NH_3_ production, as well as their impact on maize growth. The findings of this research will provide a foundation for the development of microbial biofertilizers.

## 2. Materials and Methods

### 2.1. Collection of Test Materials

Healthy *S. palustre* samples were collected in Jia Ding Village, Qiannan Buyi and Miao Autonomous Prefecture, Guizhou Province (25°55′37.55″ N, 107°38′43.13″ E, ASL: 1186.80 m) and placed in a sterile sealed bag. The samples were then placed in a foam box with an ice bag and brought back to the laboratory (College of Life Sciences, Guizhou Normal University). The plant surface of *S. palustre* was rinsed with running water to remove soil, decaying leaves, and other contaminants, and the surface moisture was then dried with sterile filter paper.

The maize seeds used in the study were HuaWan 267 variety, purchased from Anhui Longping High-tech Seed Industry, Hefei, China.

### 2.2. Culture Medium Preparation

Nutrient agar (NA) and nutrient broth (NB) media were prepared following the methods described by Gao et al. [38,39] for cultivating endophytic bacteria. An inorganic phosphate medium, for detecting bacterial phosphate solubilization ability, was prepared according to Wang’s method [40]. A milk agar medium, for detecting bacterial protease production, was prepared following Li’s method [41]. King’s B medium, for detecting bacterial lAA production, was prepared according to Bai’s method [42]. A modified King’s B (MKB) medium, Chrome Azurol S (CAS) siderophore detection medium, and detection solution were prepared following Chen’s method [43] for detecting bacterial siderophore production. Ashby’s nitrogen-free medium was prepared following the protocol of Chang et al. to assess bacterial-for-bacterial growth on a nitrogen-free medium [44]. A peptone water liquid culture medium was prepared following Chang’s method [44] for detecting bacterial NH_3_ production.

### 2.3. Isolation and Purification of Strains

Refer to Gao Xin ‘s method [38]. An amount of 2 g of *S. palustre* was weighed, rinsed three times with sterile water, soaked in 75% ethanol for 30 s, and then immersed in a hydrogen peroxide solution for 6 min. It was rinsed three times with sterile water for surface disinfection. The final rinse of sterile water (200 µL) was spread onto the NA culture medium in Section 2.2 for observation. The culture was maintained for 3 consecutive days without any microbial growth, confirming the successful surface disinfection of *S. palustre*.

Under sterile conditions, the epidermis of surface-sterilized *S. palustre* was aseptically removed. Following the protocol described by Huo et al. [45], the tissue was homogenized in a sterile mortar with 2 mL of sterile distilled water to create a uniform slurry. Then, 200 µL of the ground liquid was spread onto the NA culture medium in Section 2.2 and incubated at a constant temperature of 37 °C for 3 days. The growth of colonies was observed daily. After the colonies emerged, single colonies were picked with a sterile inoculation loop and streaked onto fresh media. This process was repeated three times until a single colony was obtained, which was designated as J11 and stored in 50% glycerol. The 16S rRNA gene sequence of this *B. cereus* strain has been deposited in the NCBI GenBank database under accession number PV465233.1.

### 2.4. Preparation of Bacterial Liquid Medium for Strain J11 

Individual colonies were picked using a sterile inoculation loop and transferred to an NB medium and incubated at 37 °C with shaking at 170 rpm/min on a shaker (Shanghai Yiheng Scientific Instrument, Shanghai, China) for 24 h. The bacterial suspension was prepared with a concentration of 2 × 10^9^ cells/mL for subsequent use.

### 2.5. Morphological Characterization of Strain J11

1. Morphological identification

A total of 1 mL from sample 2.4 was retrieved, and the bacterial suspension was diluted with sterile water to a concentration of 2 × 10^3^ cells/mL. Then, 200 µL was taken and spread onto an NA agar medium. The culture was incubated in a constant temperature incubator at 37 °C (Tianjin TESCO Instrument, Tianjin, China) for 24 h. The appearance, morphology, color, and other characteristics of the bacterial colonies were observed.

2. Gram staining

Using a 100 µL pipette, 10 µL from the 2.4 concentration of the bacterial culture was transferred and spread evenly onto a slide. The sample was allowed to air dry for 30 min, then fixed by passing it through the flame of an alcohol lamp. Gram staining was performed using a Gram staining kit (Beijing Soleibao Technology, Beijing, China). The sample was infected with 1 mL of crystal violet staining solution for 1 min, washed with distilled water until the effluent was colorless, counterstained with 1 mL iodine solution for 1 min, washed with distilled water until the effluent was colorless, washed with 1 mL decolorizing solution for 30 s, counterstained with 1 mL safranine staining solution for 1 min, washed with distilled water until the effluent was colorless, and then the results were observed under an optical microscope (Shanghai Dilun Optical Instrument, Shanghai, China). After staining, a blue–purple color indicated Gram-positive, while a red color indicated Gram-negative.

3. Spore staining

Using a 100 µL pipette, 10 µL of the culture grown for 36 h was transferred and spread evenly onto a slide. The slide was allowed to air dry for 30 min, then fixed by passing it through the flame of an alcohol lamp. Spore staining was performed using a spore staining kit (Beijing Solarbio Science & Technology, Beijing, China). A total of 1 mL of chite green staining solution was applied to the stained area on the slide, which was then heated on a microflame for 5 min. The slide was washed with distilled water until the runoff water was colorless, followed by the application of 1 mL of safranin stain for 2 min, and washed again with distilled water until the runoff water was colorless. Finally, the spore staining results were observed and photographed under an oil immersion microscope (Shanghai Dilun Optical Instrument, Shanghai, China).

### 2.6. Molecular Biology Identification of Strain J11

The colony PCR method was used to amplify the 16S rRNA gene segment of the bacterial strains (primers 27F; 1492R) [42]. The amplified PCR products were sent to Sangon Biotech (Shanghai, China) for detection. The sequences obtained were input into DNAMAN 7.0 (Lynnon Biosoft, San Ramon, CA, USA) software for sequence assembly and BLAST comparison in the NCBI database (www.ncbi.nlm.nih.gov/BLAST, accessed on 15 December 2024). Finally, the phylogenetic tree of strain J11 was constructed using MEGA 11.0 (Masatoshi Nei, USA) software, employing the maximum likelihood method with a bootstrap value set to 1000 and other values at the default settings.

### 2.7. Characterization of Physiological and Biochemical Indices for Strain J11

A hemolysis test, catalase test, starch hydrolysis test, gelatin liquefaction test, lysozyme resistance test, V-P test, citrate utilization test, and methylred test were conducted on strain J11 after 24 h of cultivation. The physiological and biochemical characteristics were primarily analyzed based on methods outlined in the “Manual for Systematic Identification of Common Bacteria” [46], “Microbiology Laboratory Manual” [47], and the analysis procedures in GB4789.14-2014 “Food Safety National Standard for Microbiological Examination of Food-*Bacillus cereus* Examination” [48].

### 2.8. Growth Curve of Strain J11

A total of 1 mL from culture 2.4 was transferred using a pipette to inoculate in a 150 mL conical flask containing 50 mL of nutrient broth (NB). The culture was incubated at 37 °C with a shaking speed of 180 rpm for 40 h. Samples were taken every 2 h, with three replicates set up for each treatment. The optical density (OD) was measured at 600 nm using a UV-visible spectrophotometer (Shanghai Youke Instrument, Shanghai, China). It was measured, and the growth curve of the strain under these conditions were plotted.

### 2.9. Functional Study of Strain J11

Phosphorus solubilization test: following the method described by Wang et al. [40,49], the strain J11 was inoculated onto the inorganic phosphate medium from Section 2.2 using sterile bamboo sticks and incubated at a constant temperature of 37 °C in an incubator for 10 days. Daily observations were made for changes in colony morphology and the appearance of a clear zone around them. A clear zone around the colony indicated that the strain had phosphorus-solubilizing ability. Starting from day 4, the diameter (D) of the clear zone and the diameter (d) of the colony were measured using a ruler, and this measurement was continued for 7 days. The ratio of clear zone diameter to colony diameter was calculated as the solubilizable phosphorus index (PSI = D/d). Each treatment was replicated three times.

A total of 1 mL from the culture 2.4 was inoculated into a 150 mL conical flask containing 50 mL of inorganic phosphate liquid medium. The flask was placed in a shaker at 37 °C with a rotation speed of 170 rpm for continuous cultivation for 7 days. Each day, 5 mL of the fermentation broth was taken, centrifuged at 4 °C with a speed of 12,000 rpm for 5 min, and the supernatant was retained. The supernatant from an inorganic phosphate culture medium without inoculation served as the control group. The soluble phosphorus concentration in the supernatant was quantified using the molybdenum-antimony anti-colorimetric method [50]. This measurement was performed daily for 7 consecutive days. Each treatment was replicated three times.

Proteinase production test: following Li Wenpung’s method [41], the strain J11 was inoculated onto the milk agar medium from Section 2.2 using sterile bamboo sticks, and incubated at a constant temperature of 37 °C for 96 h. If a clear zone appears around the colony, it indicates that the strain has the ability to produce proteinase. The diameter (D) of the clear zone and the diameter (d) of the colony were measured every 12 h, and the ratio D/d was calculated. Each treatment was replicated three times.

IAA production test: following the methods outlined by Bai et al. [42,51], 1 mL of the culture from Section 2.4 was inoculated into a 150 mL conical flask containing 50 mL of King’s liquid medium from Section 2.2, and incubated at 28 °C with a shaking speed of 180 rpm for a period of 7 days. Daily, 2 mL of the culture was taken, centrifuged at 6000 rpm for 5 min, and the supernatant was mixed with an equal volume of Salkowski color development solution (Feijing Biotechnology, Xuzhou, China). The supernatant from the un-inoculated King’s medium was mixed with the Salkowski color development solution and used as a control group. The mixture was allowed to stand in darkness for 30 min before any color changes were observed. If the mixture turned pink or red, it indicated that the strain possesses IAA production capability. The IAA production amount of strain J11 was determined using the Salkowski colorimetric method. Each treatment was replicated three times.

Siderophore production test: following the method outlined by Chen et al. [43,52], the strain J11 was inoculated onto the MKB solid culture medium from Section 2.2 using aseptic bamboo sticks and incubated at a constant temperature of 37 °C for 24 h. The CAS solid culture medium was prepared and sterilized under high pressure. Once sterilization was complete and the medium cooled to 45 °C, 15 mL of the CAS solid medium was poured into each MKB medium. The media were allowed to stand for 1 h, and any color changes around the colonies were observed. If an orange–yellow halo is observed around the colonies on the plate, it indicates that the strain possesses the ability to produce siderophore.

Growth potential testing in nitrogen-free environments: using the method described by Gong et al. [53], the strain J11 was inoculated onto the Ashby’s medium with a sterile bamboo stick and incubated at 37 °C in a constant temperature incubator for 7 days. Daily observations of bacterial growth were conducted. The formation of bacterial colonies on the medium surface indicates the strain’s potential to grow in nitrogen-free environments.

NH_3_ production test: following the method outlined by Wang and others [40,44], 200 µL of the bacterial liquid from Section 2.4 was inoculated into 10 mL of the protein broth liquid medium from Section 2.2. The mixture was cultivated at 37 °C with a shaking speed of 180 rpm for 2 days. Afterward, 0.5 mL of Nessler reagent (Shanghai Macrolin Biochemical Technology, Shanghai, China) was added. If a brownish–yellow precipitate appears, this indicates that the strain has the ability to produce NH_3_.

### 2.10. Strain J11’s Promoting Test on Maize

#### 2.10.1. Germination Test

Bacterial suspension preparation: 40 mL of the bacterial culture from Section 2.4 was transferred to a 50 mL centrifuge tube and centrifuged at 6000 rpm at 4 °C for 5 min. The supernatant was discarded, and 20 mL of sterile water was added to the bacterial cell layer. After a second centrifugation, the supernatant was discarded. This process was repeated three times, resulting in a bacterial suspension with a concentration of 2 × 10^9^ cells/mL. The suspension was then diluted with sterile water to six different concentrations: one-fold (2 × 10^9^ cells/mL), two-fold (1 × 10^9^ cells/mL), four-fold (5 × 10^8^ cells/mL), eight-fold (2.5 × 10^8^ cells/mL), sixteen-fold (1.25 × 10^8^ cells/mL), and thirty-two-fold (6.25 × 10^7^ cells/mL) for future use.

Seed sterilization: 20 plump and uniformly sized Huawan 267 maize seeds were selected [54]. The sterilization of these corn seeds was carried out following the stepwise chemical sterilization method using 75% alcohol and 2% sodium hypochlorite [54,55,56].

Seed soaking: the soaking of maize seeds was conducted using the method described by Zhao et al. [57]. The disinfected maize seeds were individually immersed in six gradient concentrations of the J11 bacterial suspension, diluted 1, 2, 4, 8, 16, and 32 times in 100 mL volumes, respectively. In order to quickly break the seed dormancy and shorten the germination cycle, the seeds were immersed at room temperature for 8 h [57]. A control group was treated with 100 mL of sterile water for sowing. All other conditions were kept consistent with the experimental group.

Cultivation: in the experimental group, following the method described by Xiong et al. [58], 20 seeds soaked in the bacterial solution were placed in a germination box (12 × 12 × 5 cm^3^) lined with a layer of filter paper, arranged in four rows with five seeds per row. Each treatment was replicated three times. For the control group, seeds soaked in the sterile water were utilized, while all other conditions were kept identical to the experimental group. The seeds were continuously cultivated for 2 days in a growth chamber (Hefei Huadeli Scientific Equipment, Hefei, China) maintained at a temperature of 28 ± 1 °C, relative humidity of 70 ± 5%, and a light cycle of L:D = 12:12. The number of germinated seeds was observed daily during the cultivation period. The penetration of the germ through the seed coat is taken to be the sign of germination [59]. On the second day, the length of the primary root and bud of the maize seedlings was measured using a vernier caliper, and the number of secondary roots was recorded. Germination rate, germination index, and other related parameters were calculated.

Germination rate (%) = (total number of germinated seeds/total number of test seeds) × 100%.

Growth Index (GI) = ∑(Gt/Dt), where Gt is the number of germinated seeds on the t-th day, and Dt is the corresponding germination days.

#### 2.10.2. Houseplant Experiment

The maize seeds were disinfected as described in Section 2.10.1 and placed in a constant temperature incubator at 28 °C for 24 h, until they germinated and were ready for use. Peat soil with a particle size of 0-6 mm (Pindstrup Mosebrug A/S, Ryomgard, Denmark) and vermiculite with a particle size of 1–3 mm (Shijiazhuang Red Grass Trading, China) were mixed in a 1:1 volume ratio and then sterilized at 121 °C for 30 min [60]. A 50.00 g portion of the mixture was placed into a seedling tray (6.5 × 6.5 × 7.0 cm^3^). The soil was wetted with 200 mL of sterile water, and one germinated seed was planted per pot at a depth of approximately 2 cm. The control group was irrigated with 5 mL sterile water at 6:00 p.m. every day, and the experimental group was irrigated with 5 mL of a bacterial suspension diluted 16-fold in a 2.10.1 dilution system at 6:00 p.m. every day for 7 consecutive days. From the 8th to the 10th day, both the experimental group and the control group were irrigated with 5 mL of sterile water at 6:00 p.m. daily. Each treatment was replicated three times. The height of each plant and leaf length were measured using a ruler on the tenth day after the experiment began. A Yield Meter (Zhejiang Toppan Yunnong Science and Technology, Hangzhou, China) was used for measuring leaf width, a Leaf Chlorophyll Meter (Zhejiang Toppan Yunnong Science and Technology, Hangzhou, China) for determining the relative chlorophyll content in leaves (SPAD value), and an Ultra-Micro Electronic Analytical Scale (Shanghai Jingqi Instrument, Shanghai, China) for measuring above-ground and below-ground fresh weights.

### 2.11. Data Analysis

In this study, basic data statistical analysis was conducted using Microsoft Excel 2016. For statistical analysis, IBM SPSS version 26.0 was utilized. The independent samples t-test method was applied at a significance level of 0.05 to analyze the difference in two groups of data. For multiple group data, the Tukey method was employed at the same significance level for difference significance analysis. Graphs were created using software MEGA 11.0 and GraphPad Prism 8.0.

## 3. Results and Analysis

### 3.1. Identification of Endophytic Bacteria J11 Within S. palustre

#### 3.1.1. Morphological Identification

**Figure 1:**
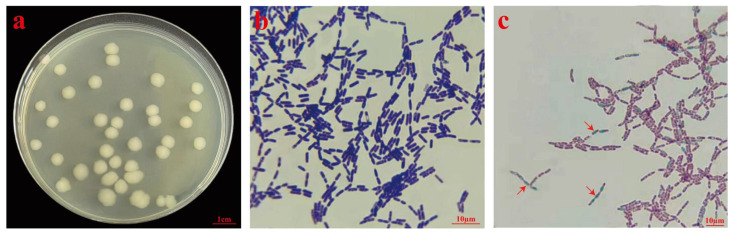
Morphological identification of strain J11. Note: (**a**): colony morphology of strain J11; (**b**): strain J11 Gram staining (100×); (**c**): spore staining of strain J11 (100×).

After 24 h of cultivation, the colony of strain J11 appeared milky white, with a rough, opaque surface, a raised center, and irregular edges, with a diameter of 5.50 ± 0.53 mm (Figure 1a). The cells were rod-shaped, measuring 1.0–1.1 µm × 3.0–4.7 µm (Figure 1b), appearing either singularly or in long chains. Gram staining showed a blue–purple color (Figure 1b), indicating that strain J11 was a Gram-positive bacterium. After spore staining, the spores appeared green and clearly visible, oval in shape, located centrally or at the ends of the bacterial cells (Figure 1c). These characteristics are consistent with those of *B. cereus*; thus, the strain J11 was preliminarily identified as *B. cereus.*

#### 3.1.2. Molecular Biology Identification

**Figure 2:**
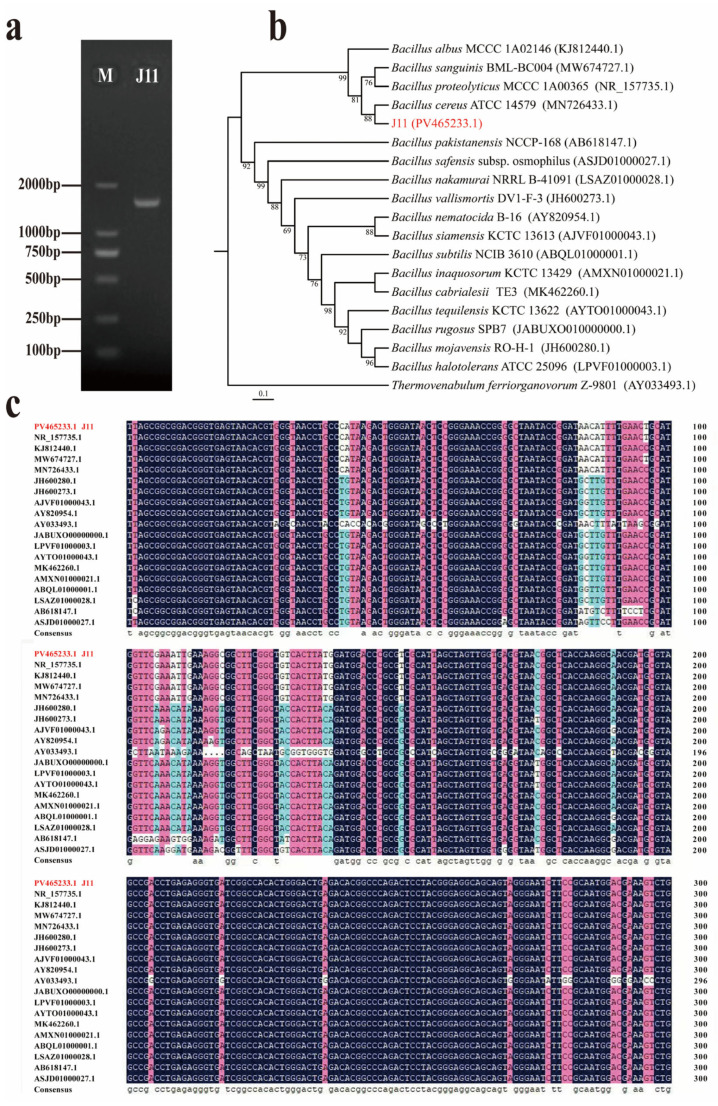
Molecular biological identification of strain J11. Note: (**a**): gel electrophoresis map; (**b**): phylogenetic tree of strain J11; (**c**): DNAMAN homology comparison map of strain J11.

The 16S rRNA gene sequence of strain J11 has a length of 1462 bp (Figure 2a). A BLAST search was performed in the NCBI database, and a phylogenetic tree was constructed using MEGA 10.0 software (Figure 2b). The phylogenetic tree demonstrated that strain J11 (PV465233.1) clusters with *B. cereus* ATCC 14579 (MN726433.1), forming a distinct clade supported by an 88% bootstrap value. This finding is consistent with the results of DNAMAN sequence alignment (Figure 2c). Based on both morphological and molecular biological identification, strain J11 was ultimately determined to be *B. cereus*.

### 3.2. Physiological and Biochemical Characteristics of Endophytic Bacteria J11 Within S. palustre

**Figure 3:**
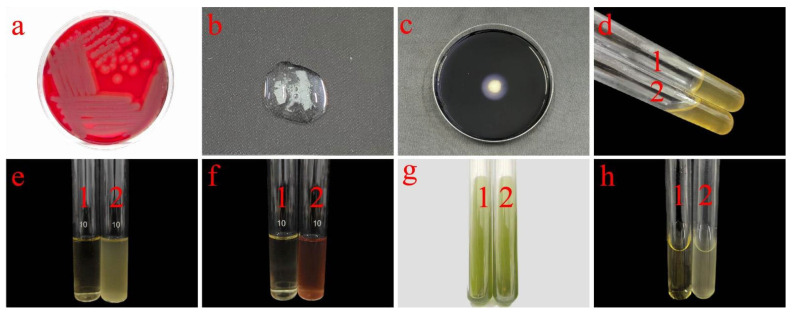
Phenomena of physiological and biochemical tests for strain J11. Note: (**a**): hemolysis test; (**b**): catalase test; (**c**): starch hydrolysis test; (**d**): gelatin liquefaction test; (**e**): lysozyme resistance test; (**f**): V-P test; (**g**): citrate utilization test; (**h**): methylred test; in figures (**d**–**h**), (**1**) represents control group, and (**2**) represents experimental group.

As shown in Table 1 and Figure 3, strain J11 tested negative in the methylred and citrate utilization tests, while it tested positive in the hemolysis, catalase, starch hydrolysist, gelatin liquefaction, V-P, and lysozyme tests. All physiological and biochemical characteristics were consistent with those of *B. cereus* [61]. Therefore, based on both morphological characteristics and comparison with the *Berger’s Manual of Systematic Microbiology* and *Berger’s Manual of Classification of Bacteria* [62], the strain was further confirmed to be a *B. cereus*.

### 3.3. Growth Curve Analysis of Strain J11

**Figure 4:**
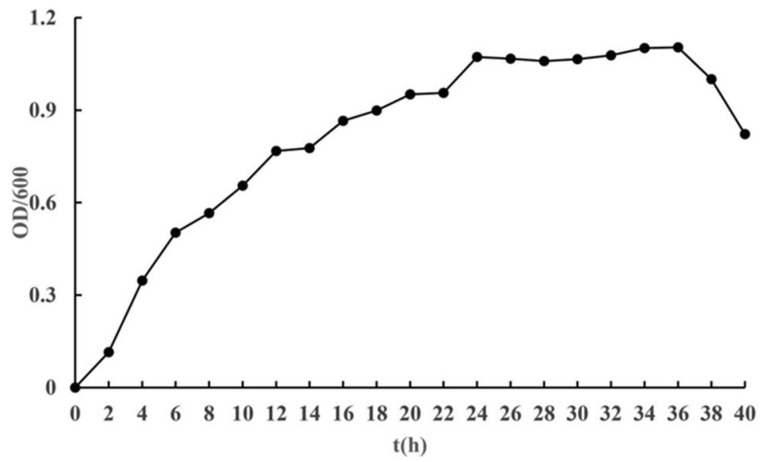
Growth curve of strain J11.

The bacterial growth cycle is typically divided into four distinct phases: the lag phase, the log (logarithmic) phase, the stationary phase, and the death phase [63]. However, the duration of each stage is contingent upon multiple factors. These include the specific growth environment, the composition and properties of the culture medium (such as the presence of pigments in the medium), and the inherent physiological characteristics of the microorganisms, which are genetically determined [64]. As illustrated by the growth curve of strain J11 (Figure 4), strain J11 began growing at time point 0, with an increase in OD_600_ value over time followed by a decrease. From 0 to 2 h, the growth was slow, indicating the lag phase; from 2 to 24 h, the growth was rapid, following a logarithmic increase, which corresponds to the log phase; from 24 to 36 h, the growth rate plateaued, indicating the stationary phase; after 36 h, the growth sharply declined, entering the death phase. Furthermore, since bacterial seed cultures in production are typically selected during the log phase, this stage provides both vigorous proliferative capacity and high concentration, thereby shortening the fermentation cycle [65,66,67].

### 3.4. Functional Analysis of Strain J11

**Figure 5:**
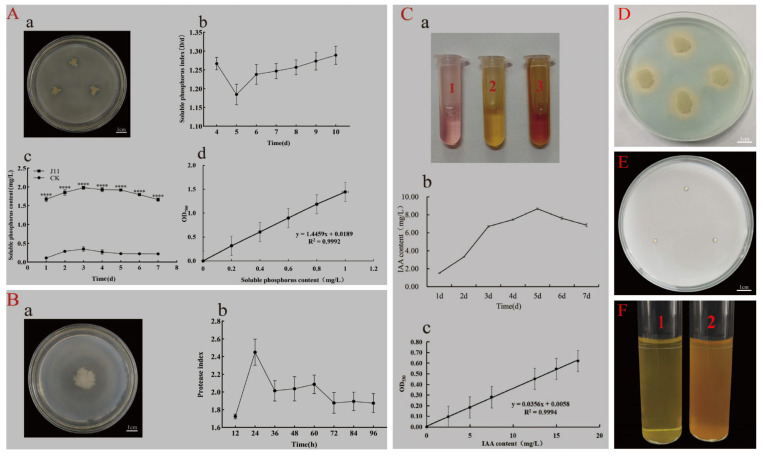
Functional characteristics of strain J11. Note: (**A**): test for phosphate-solubilizing ability; (**A**(**a**)): dissolved inorganic phosphorus colony front, 10 d; (**A**(**b**)) represents the solubility index of phosphorus for the J11 strain from 4 to 10 d; (**A**(**c**)) represents the amount of phosphorus released by the J11 strain from 1 to 7 d; (**A**(**d**)) represents the standard curve of soluble phosphorus; (**B**): test for protease production ability, (**B**(**a**)): protease-producing colony front, 96 h; (**B**(**b**)) represents the index of protein degradation by the J11 strain from 12 h to 96 h. (**C**): test for IAA production ability, where (**C**(**a1**)) is the standard sample of 5 mg/L IAA, (**C**(**a2**)) is the control group, and (**C**(**a3**)) is the experimental group at day 5; (**C**(**b**)) represents the amount of auxin produced by the J11 strain from day 1 to day 7; (**C**(**c**)) represents the standard curve for IAA. (**D**): test for iron carrier production capacity; (**E**): test for growth potential in nitrogen-free environments; (**F**): test for NH₃ production capacity. (**Fl**) the control group; (**F2**) the experimental group; ****: *p* < 0.0001.

On an inorganic phosphate medium, strain J11 formed a clear zone around its growth (Figure 5A(a)), indicating its phosphate solubilization ability. The phosphate solubilization index (PSI) showed a trend of first decreasing and then increasing as the cultivation time progressed (Figure 5A(b)), reaching its lowest value of 1.18 ± 0.08 on day 5 and the highest value of 1.30 ± 0.07 on day 10. This might be due to the strain’s adaption to the new environment during its initial growth phase, where the phosphate-solubilizing ability was weaker, resulting in a lower PSI. As the cultivation time increased, the phosphate-solubilizing bacteria gradually adapt, multiply in large numbers, and actively decompose inorganic phosphates, leading to an increase in the PSI until it reached its peak. The trend of increasing phosphate solubilization is followed by a decrease as cultivation time increases, with all experimental groups showing higher values than the control group by factors ranging from 5.66 to 15.18 times (Figure 5A(c)). The maximum amount of phosphate solubilized occurred on day 3 at a value of 1.98 ± 0.07 mg/L and subsequently decreased due to the high nutrient content in the liquid culture medium during the initial phase; as bacterial concentration increases, soluble phosphorus content also rises [68]. The pH value of the control group’s culture medium was recorded as 6.14 ± 0.10 on day 7 (Figure 5A(d)), while that of the experimental group’s fermentation broth was at a level of 4.69 ± 0.08 on day 7 (Figure 5A(d)). This suggests that organic acids are produced by the microorganisms during their growth process. The speculation about their phosphorus-solubilizing mechanism is based on their ability to lower environmental pH through production of organic acids, which facilitates dissolution or chelation of insoluble mineral phosphates [69,70]. The standard curve for soluble phosphorus is shown in Figure 5A(d) with a linear regression equation of y = 1.4459X + 0.0189 and R^2^ = 0.9992; indicating a good linear correlation between the two.

On the milk agar medium, strain J11 formed a clear zone around its growth (Figure 5B(a)), confirming its protease-production ability. The protease solubilization index showed an increase followed by a decrease over time, reaching a maximum of 2.45 ± 0.26 at 24 h, after which the index fluctuated and decreased, stabilizing at 72 h (Figure 5B(b)). This is because the strain is metabolically active in the early stages of growth, producing large amounts of protease to degrade protein. However, as the protein is consumed, the strain’s metabolism slows down, resulting in the fluctuating decrease in the solubilization index [71].

The strain was cultured in King’s medium under suspension culture conditions, and the addition of the colorimetric solution turned the medium red (Figure 5C(a1–a3)), indicating that strain J11 is capable of producing IAA. The IAA production showed a trend of first increasing and then decreasing over time. As tryptophan was consumed, the IAA content increased and reached its maximum value of 8.67 ± 0.16 mg/L on day 5. Once the nutrients, including precursors for IAA synthesis, were exhausted, IAA was gradually degraded by indoleacetic acid oxidase, leading to a decrease in IAA levels (Figure 5C(b)). Figure 5C(c) shows the standard curve for IAA, with the linear regression equation y = 0.0356X + 0.0058 and R^2^ = 0.9994, indicating a strong linear correlation between the two.

On the CAS detection medium, strain J11 formed an orange–yellow halo around its growth, indicating its ability to produce siderophores (Figure 5D). On the Ashby medium, the J11 strain formed irregular oval colonies. These colonies had a yellow center surrounded by a white periphery (Figure 5E). Considering the selectivity of the nitrogen-free Ashby medium, this observation suggests that the J11 strain potentially has the ability to grow in a nitrogen-free environment [53]. Furthermore, after adding Nessler colorimetric solution, the bacterial suspension turned into a brownish yellow color (Figure 5F2) compared to a yellow color in the control group (Figure 5F1), suggesting that strain J11 has the ability to produce NH_3_.

In conclusion, strain J11 is identified as a multifunctional strain. It possesses the capabilities of solubilizing phosphate, producing protease, indole-3-acetic acid (IAA), siderophores, and NH_3_. These characteristics clearly demonstrate its potential to promote plant growth.

### 3.5. The Effect of Strain J11 on the Growth of Maize Seeds and Seedlings

#### 3.5.1. Effect of Strain J11 on Maize Seed Germination

**Figure 6:**
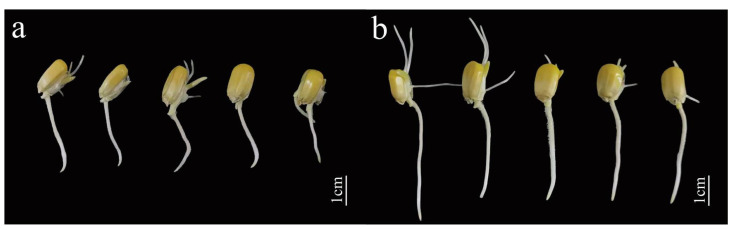
Effect of J11 strain on maize embryo roots and buds. Note: (**a**): control group; (**b**): experimental group (diluted 16 times of J11 bacterial suspension).

**Figure 7:**
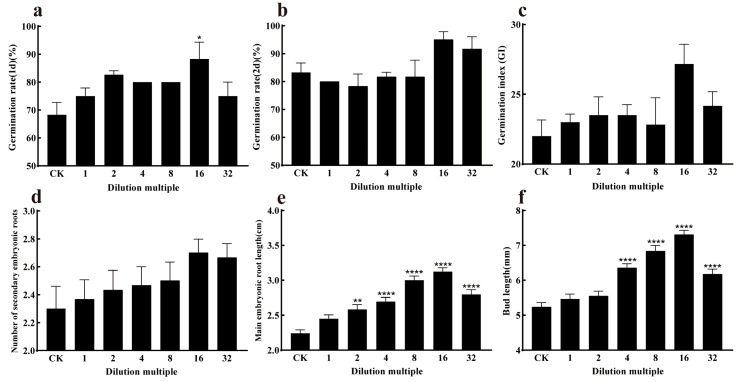
Effect of the J11 strain on maize seed germination. Note: (**a**) represents the germination rate of maize seeds on the first day; (**b**) represents the germination rate of maize seeds on the second day; (**c**) represents the germination index of maize seeds; (**d**) represents the number of radicles; (**e**) represents the length of the primary embryo root; (**f**) represents the bud length of the maize seed; *: *p* < 0.05; **: *p* < 0.01; ****: *p* < 0.0001.

The strain J11 was diluted at different concentrations and then used to soak maize seeds. On day 1, the germination rates of maize seeds treated with different dilutions of strain J11 were significantly higher than those of the control group (*p* < 0.05), they were 75.00 ± 0.88%, 82.66 ± 1.45%, 80.00 ± 0.00%, 80.00 ± 0.00%, 88.33 ± 6.00%, and 75.00 ± 5.00%, respectively, with increases of 9.76%, 19.12%, 17.08%, 17.08%, 29.27%, and 9.76%, respectively. This indicates that the suspension of strain J11 has a positive effect on enhancing the germination of maize seeds. The germination of the seeds exhibited a stimulatory effect. Among the treatments, the 16-fold diluted bacterial suspension resulted in the highest (Figure 6 and Figure 7a), indicating that the bacterial suspension of J11 has a concentration-dependent effect on maize seed germination, with optimal efficacy observed at a 16-fold dilution. On day 2, the highest germination rate and germination index were observed with the 16-fold diluted bacterial suspension, at 95.00 ± 5.00% and 27.17 ± 2.47, respectively (Figure 7b,c); although, these values were not significantly different from the control (*p* > 0.05). This might be due to its high seed vigor. Additionally, on the second day, there was no significant difference in the number of secondary roots compared to the control group (*p* > 0.05) (Figure 7d); this indicates that, on the second day after soaking, the J11 bacterial strain has no significant impact on the germination rate, germination index, and secondary root number of maize plants. This finding implies that, under consistent environmental conditions, the germination of plant seeds and moss plant spores mainly relies on stored nutrients [72]. The lack of significant influence of J11 bacterial suspension on the second day’s maize seed germination in this study might be attributed to maize seeds consuming a large amount of nutrients during their first day’s germination, leading to depletion of stored nutrients and consequently no significant influence on subsequent days’ seed germination.

The differences in the root and bud lengths of the experimental groups compared to the control groups were significantly pronounced (*p* < 0.05) under various dilutions, as shown in Figure 7e,f. The root lengths were 2.45 ± 0.05 cm, 2.58 ± 0.07 cm, 2.69 ± 0.06 cm, 3.00 ± 0.06 cm, 3.12 ± 0.05 cm, and 2.79 ± 0.06 cm, respectively. They increased by 9.37%, 13.87%, 20.08%, 33.92%, 39.28%, and 25.00% with each successive dilution. This indicates that the root length gradually increased, reaching a peak at a 16-fold dilution and then decreasing subsequently. The bud lengths were 5.46 ± 0.13 mm, 5.54 ± 0.14 mm, 6.35 ± 0.11 mm, 6.83 ± 0.16 mm, 7.30 ± 0.12 mm, and 6.17 ± 0.14 mm, respectively. They increased by 4.58%, 5.85%, 21.41%, 30.78%, 39.77%, and 18.16% with each successive dilution. This indicates that the root length gradually increased, reaching a peak at a 16-fold dilution and then decreasing subsequently. These results suggest that the J11 bacterial suspension influences root and bud growth, such as by producing growth hormones (or growth factors). The effect is significant and exhibits a concentration-dependent pattern, which is consistent with the findings of Ha et al. [73,74]. Both lower and higher concentrations of growth hormones can inhibit growth. In this study, strain J11 promotes maize root and bud growth, possibly due to its production of IAA, which accelerates the developmental process [75,76].

The results indicate that strain J11 has a concentration-dependent effect on maize germination rate, main radicle length, and bud length, and that the specific influence mechanism needs to be further explored.

#### 3.5.2. Effect of Strain J11 on the Growth of Maize Seedlings

**Figure 8:**
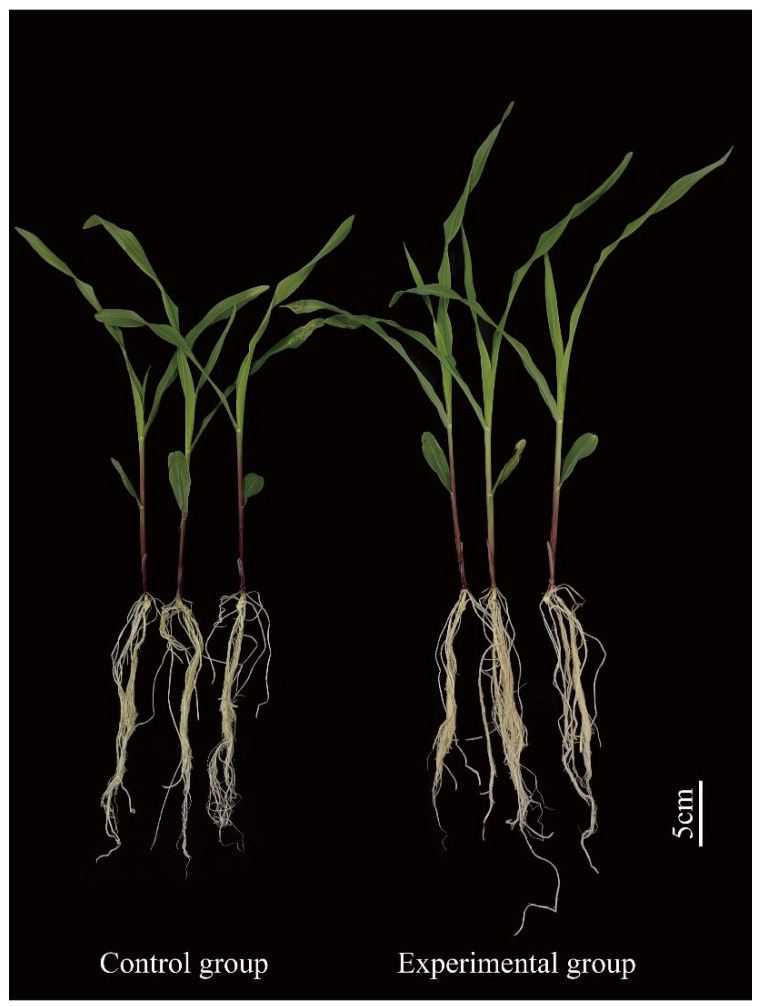
Effect of strain J11 on maize seedlings.

**Figure 9:**
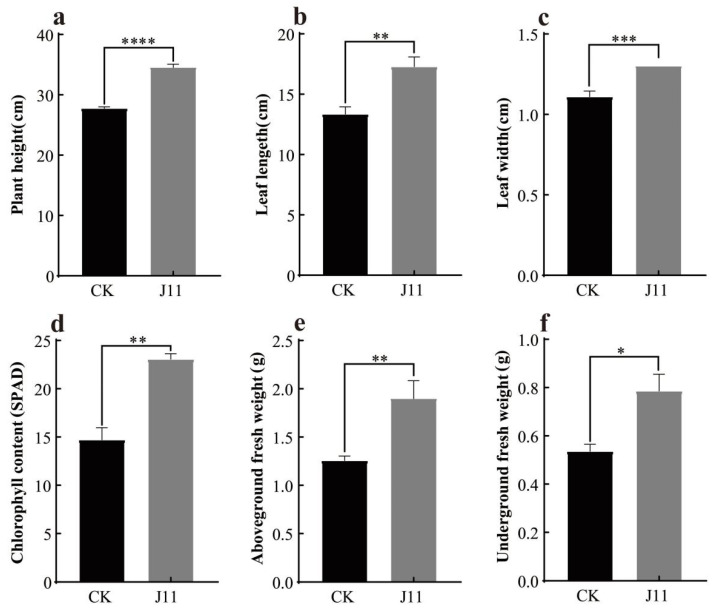
Promoting effect of strain J11 on maize seedlings. Note: (**a**): effect on maize plant height; (**b**): effect on maize leaf length; (**c**): effect on maize leaf width; (**d**): effect on chlorophyll content of maize leaves; (**e**): effect on above-ground fresh weight of maize; (**f**): effect on below-ground fresh weight of maize; *: *p* < 0.05; **: *p* < 0.01; ***: *p* < 0.001; ****: *p* < 0.0001.

As shown in Figure 8, following the root application of J11 bacterial liquid, the plant height, leaf length, and leaf width of maize exhibited a highly significant increase (*p* < 0.001). The values were 34.53 ± 0.31 cm, 17.29 ± 0.45 cm, and 1.30 ± 0.00 cm, respectively, which increased by 24.21%, 17.12%, and 29.51%, respectively (Figure 9a, Figure 9b, Figure 9c). This might be attributed to J11’s ability to solubilize phosphorus, produce protease, siderophore, and NH_3_ for promoting nutrient absorption in maize and stimulating its growth development [77], although the specific mechanisms require further investigation. Beyond growth indicators, the chlorophyll content of maize treated with strain J11 bacterial suspension also showed a significant increase. This phenomenon may be attributed to the fact that the J11 strain enhanced the absorption of phosphorus and other nutrients in maize seedlings [78,79]. This, in turn, accelerated the synthesis of substances such as DNA, RNA, and ATP. As a result, the accumulation of chlorophyll was promoted, leading to an increase in the chlorophyll content of seedling leaves. The chlorophyll content reached 23.06 ± 0.57 spad, representing a 56.81% increase (Figure 9d). The enhanced chlorophyll content augmented photosynthesis. Subsequently, the accumulation of photosynthetic products led to an increase in both the above-ground fresh weight and the underground fresh weight. The above-ground fresh weight was 1.90 ± 0.10 g, showing a 50.79% increase, and the underground fresh weight was 0.78 ± 0.06 g, with a 46.30% increase (Figure 9e,f).

## 4. Discussion

Endophytic bacteria are ubiquitously found within the *Sphagnum*, establishing a mutualistic relationship with the host over long evolutionary periods [21,80]. *B. cereus* is capable of producing plant growth hormones, transforming organic nitrogen and phosphorus, degrading insoluble inorganic salts, and enhancing nutrient absorption in plants, thus promoting plant growth [81,82]. In previous studies, *B. cereus* has been isolated from various sources such as watermelon leaves [83], rice [84], cattle and horses [30], fish [85], termites [86], and soil [87,88]. In this study, *B. cereus* strain J11 was isolated from *Sphagnum* for the first time through morphological characterization, physiological and biochemical assays, and 16S rRNA gene sequence analysis. Based on these findings, it was identified as an endophytic bacterium of *Sphagnum*, thereby expanding the known diversity of endophytic *Bacillus* species. Future research could further elucidate the taxonomic position and evolutionary relationships of this strain through comprehensive molecular genetic analyses.

The growth cycle of bacteria typically comprises four phases: lag, log, stationary, and death. The duration of these phases can vary for different growth phases of the same bacterial strain depending on the specific strain or the nutrient medium [63,64]. In Yan’s study [65], *B. cereus* B-21 enters the log phase between 3 and 16 h, followed by a stationary phase from 16 to 24 h, transitioning into a death phase after 24 h. Zhou’s research [66] indicates that the log phase for *B. cereus* lasts from 2 to 16 h, with a stationary phase from 18 to 20 h, progressing to a death phase after 22 h. Chang’s study [44] shows that the log phase for *B. cereus* spans from 6 to 22 h, followed by a stationary phase from 22 to 28 h, and it progresses into a decline phase after reaching its maximum concentration at around 30 h. Both Yan and Chang utilized the LB medium for cultivation. In contrast, this study employed the NB medium, with the log phase occurring between 2 and 24 h, the stationary phase lasting from 24 to 36 h, and the death phase occurring after the maximum concentration was reached at 36 h. In comparison to the aforementioned studies, the logarithmic and stationary phases in this study are relatively long. When contrasted with *B. cereus* B-21, the growth cycle of the strain in this study is longer [65], which is due to the differences in the culture medium and the strains themselves. Such findings offer valuable insights for further research on the industrial production of *B. cereus*.

*B. cereus* possesses numerous potential growth-promoting functions and is an important plant growth-promoting bacterium (PGPB) [89]. Kulkova et al. found that *B. cereus* is capable of producing IAA, aminocyclopropane-1-carboxylic acid (ACC) deaminase, and phosphate solubilization [89]; Bizani et al. found that *B. cereus* possesses the ability to produce proteases, amylases, antibiotics, and cellulases among other active substances [90,91]; Sherpa et al. discovered that *B. cereus* has the ability to produce IAA, siderophores, and phosphate solubilization abilities [92]; Khan et al. found that *B. cereus* SAl can produce IAA, gibberellins, and organic acids [93]. In this study, *B. cereus* J11 exhibits the abilities of phosphorus solubilization, as well as proteases, IAA, siderophore, and NH_3_ production. In comparison with the *B. cereus* strain investigated in the aforementioned study, the *B. cereus* J11 strain in the present study additionally has the capacity to produce NH_3._ This characteristic renders it a promising bio-stimulant for crop growth. This highlights its potential as a valuable microbial resource for sustainable agricultural development.

*B. cereus* can promote the growth of various crops [89,94,95]. In a study by Sherpa et al., *B. cereus* strain P8 was found to increase the above-ground biomass and root system of pea plants [92]. Ali et al.’s research indicates that *B. cereus* can enhance potato plant height, stem dry weight, and nutrient concentrations (N, P, K) in leaves [96]; Ku et al.’s study shows that *B. cereus* strain YL6 promotes the growth of soybeans and wheat, increasing the total phosphorus content in leaf tissue [97]; Baliyan et al.’s research demonstrates that *B. cereus* strain MEN8 can increase the length and weight of roots and sprouts in chickpea plants [98]; Ibrahim et al.’s study reveals that *B. cereus* GGBSU-I can elevate the rice germination rate and chlorophyll content [99]; Adeleke et al.’s research shows that *B. cereus* strain T4S can enhance the main root length, root length, root number, root weight, seed weight, and bud weight of sunflower plants [100]; Kumar et al.’s study indicates that *B. cereus* strain LPR2 promotes maize plant growth, including root and bud growth as well as fresh and dry weights [101]; Mukhtar et al.’s research suggests that *B. cereus* KTES strains can increase the stem and root lengths of tomato plants, along with stem and root weights as well as leaf area [102]. The *B. cereus* strains examined in the aforementioned studies all promoted crop growth and increased chlorophyll content to varying extents. This is generally consistent with the experimental findings of the present study. The *B. cereus* used in this study facilitates maize seed germination and enhances it. The maize seedling height, leaf length, leaf width, above-ground fresh weight, below-ground fresh weight, and chlorophyll content are significantly higher compared to other studies with *B. cereus*. This finding lays a solid foundation for further investigations into the bio-stimulating mechanisms of *B. cereus*.

## 5. Conclusions

This study represents the first isolation of *B. cereus* from *S. palustre*, with subsequent characterization of its growth dynamics, functional properties, and growth-promoting effects on maize. This research provides valuable microbial resources and a theoretical foundation for both the development of plant growth-promoting bacteria and the functional exploration of microbial assets.

## Figures and Tables

**Table 1:** Physiological and biochemical results of strain J11. Note: +: positive; −: negative.

Test Names	Experimental Phenomenon	Results
Hemolysis test	Colonies surrounded by a completely transparent hemolytic ring (beta hemolysis)	+
Catalase test	Bubble	+
Starch hydrolysis test	With colorless transparent ring	+
Gelatin liquefaction test	The medium is liquid	+
Lysozyme resistance test	The medium becomes cloudy	+
V-P test	The culture medium is red	+
Citrate utilization test	The medium is green in color	−
Methylred test	The reaction solution is yellow	−

## Data Availability

The data that support the findings of this study are available from the corresponding author and the first author upon reasonable request.
